# Comparative Analysis of Dental Pulp and Periodontal Stem Cells: Differences in Morphology, Functionality, Osteogenic Differentiation and Proteome

**DOI:** 10.3390/biomedicines9111606

**Published:** 2021-11-03

**Authors:** Anastasia V. Kotova, Arseniy A. Lobov, Julia A. Dombrovskaya, Valentina Y. Sannikova, Nadezhda A. Ryumina, Polina Klausen, Alexey L. Shavarda, Anna B. Malashicheva, Natella I. Enukashvily

**Affiliations:** 1 Institute of Cytology of the Russian Academy of Sciences, 194064 St. Petersburg, Russia; anastkotova@gmail.com (A.V.K.); arseniylobov@gmail.com (A.A.L.); valent.sannikova@gmail.com (V.Y.S.); polina.klauzen@gmail.com (P.K.); amalashicheva@gmail.com (A.B.M.); 2 Cell Technologies Laboratory, General Dentistry Department, North-Western State Medical University, 191015 St. Petersburg, Russia; Yuliya.Dombrovskaya@szgmu.ru; 3 Cell Technologies Center Pokrovsky, LLC, 199106 St. Petersburg, Russia; nadiy007@yandex.ru; 4 Research Resource Center Molecular and Cell Technologies, Saint-Petersburg State University, 199034 St. Petersburg, Russia; shavarda@binran.ru

**Keywords:** dental stem cells, dental pulp stem cells, periodontal ligament stem cells, osteogenic differentiation, odontoblastic differentiation, pluripotency, somatic OCT4, SSEA-4, proteomics, mass-spectrometry

## Abstract

Dental stem cells are heterogeneous in their properties. Despite their common origin from neural crest stem cells, they have different functional capacities and biological functions due to niche influence. In this study, we assessed the differences between dental pulp stem cells (DPSC) and periodontal ligament stem cells (PDLSC) in their pluripotency and neuroepithelial markers transcription, morphological and functional features, osteoblast/odontoblast differentiation and proteomic profile during osteogenic differentiation. The data were collected in paired observations: two cell cultures, DPSC and PDLSC, were obtained from each donor. Both populations had the mesenchymal stem cells surface marker set exposed on their membranes but differed in *Nestin* (a marker of neuroectodermal origin) expression, morphology, and proliferation rate. *OCT4* mRNA was revealed in DPSC and PDLSC, while OCT4 protein was present in the nuclei of DPSC only. However, transcription of *OCT4* mRNA was 1000–10,000-fold lower in dental stem cells than in blastocysts. DPSC proliferated at a slower rate and have a shape closer to polygonal but they responded better to osteogenic stimuli as compared to PDLSC. *RUNX2* mRNA was detected by qPCR in both types of dental stem cells but RUNX2 protein was detected by LC-MS/MS shotgun proteomics only in PDLSC suggesting the posttranscriptional regulation. *DSPP* and *DMP1*, marker genes of odontoblastic type of osteogenic differentiation, were transcribed in DPSC but not in PDLSC samples. Our results prove that DPSC and PDLSC are different in their biology and therapeutic potential: DPSC are a good candidate for osteogenic or odontogenic bone-replacement cell-seeded medicines, while fast proliferating PDLSC are a prospective candidate for other cell products.

## 1. Introduction

A unique group of stem cells, isolated from dental and periodontal tissues, exhibit self-renewal, and multilineage differentiation capacity. These cells exhibit properties similar to bone marrow stem/stromal cells, the ‘gold standard’ of mesenchymal stromal cells (MSC) [1,2]. A specific feature that distinguishes tooth stem cells from MSC is their neuroectodermal origin. These cells originating from migrating neural crest cells represent a multipotent cell population derived from the lateral ridges of the neural plate during craniofacial development [3]. During embryogenesis, the progenitor cells migrated from the neural crest are involved in the formation of tooth buds. The neural crest is also referred as the fourth germ layer, because many organs and tissues are formed from its cells. Cells migrating from the neural crest can’t be called “stem” in the strict sense of the word, since the properties related to stemness are more limited in these cells than in ‘true’ stem cells. However, they are multipotent. Dental stem cells are heterogeneous in their properties as neural crest ‘stem’ cells. Tooth pulp contains various types of cells such as endothelial cells, neurons, fibroblasts, osteoblasts, osteoclasts, odontoblasts as well as cells that are referred as postnatal dental pulp stem cells (DPSCs). Also, four more types of dental stem cell-like populations were identified and characterized: stem cells from human exfoliated deciduous teeth (SHED), periodontal ligament stem cells (PDLSC), stem cells from the apical papilla (SCAP), and population of dental follicle-derived progenitor cells (DFPCs) [1]. It has been hypothesized that different populations of oral cavity-derived stem cells have different physiological features. The differences in cell biology arise due to the specific neighborhood surrounding different populations of dental stem cells.

Colonies grown from single cells have different proliferative and differentiation potentials [1,4,5,6]. In addition, some cells express a number of pluripotent markers, such as OCT4, SSEA-4, Nanog, and some others, which in embryonic cells are involved in maintaining their proliferative activity and undifferentiated state [7,8,9]. There is a possibility that a subpopulation of the progenitor cells of the neural crest, expressing these markers, persists after migration and formation of tissues in adult tissues and organs, preserving their stem potential. However, there is no direct evidence of this hypothesis yet.

Dental stem cells are capable of multilineage differentiation. They have three MSC ‘classical’ differentiation capacities (osteogenic, adipogenic, and chondrogenic) along with the cranial neural crest progenitor cells ones: they give rise to neurons, myoblasts, and odontoblasts [1,4,10,11,12,13]. It has turned out that dental stem cells are able to differentiate rather into odontoblasts than into osteoblasts under the influence of osteogenic factors [11,14,15,16]. The ability for odontoblastic differentiation is one of the most important features of pulp stem cells. A striking feature of pulp stem cells is their ability to form a complex resembling a tooth when transplanted into immunosuppressed mice cells with hydroxyapatite/tricalcium phosphate [17,18]. Transplanted cells form a vascularized pulp-like tissue surrounded by a layer of odontoblast-like cells expressing dentin sialophosphoprotein (DSPP). The tissue produces dentin-containing dentinal tubules, similar to natural dentin. If DPSC are seeded onto dentin, some of them turn into odontoblast-like cells with a polarized cell body [16,19]. However, the proteomic analysis of dental stem cells before and after osteogenic differentiation is far from being complete.

Among all dental stem cells, DPSC and PDLSC are obtained most easily: teeth are often extracted during dental treatment or maxillofacial surgery and the periodontal ligament is usually attached to an extracted tooth. Thus, DPSC and PDLSC can be obtained from medicinal waste tissues. The populations can be easily separated at the first step of cells isolation. DPSC and PDLSC differ in their biology and hence, probably, in their therapeutic potential. However, to prove that, studies on paired samples (i.e., different tissues from the same donors) are necessary.

The aim of this work was to assess the differences between DPSC and PDLC in their pluripotency and neuroepithelial markers transcription, morphological and functional features, osteoblast/odontoblast differentiation markers, and proteomic profile during osteogenic differentiation. The data were collected for paired samples: two cell cultures, DPSC and PDLSC, were obtained from each donor.

According to our data, both populations had the MSC surface marker set exposed on their membranes. At the same time, *NES* RNA, a marker of neuroectodermal origin, was revealed by qPCR in DPSC but not in PDLSC. OCT4 protein was present in the nuclei of DPSC and PDLSC, while *OCT4* mRNA was revealed in DPSC and PDLSC total RNA. However, transcription of *OCT4* mRNA was 1000–10,000-fold lower in dental stem cells than in blastocysts. The low level of *OCT4* transcription combined with the data about the low intensity of the nuclear staining led to a suggestion that it does not function as a pluripotency maintaining transcription factor but plays a different role in dental stem cells. Nevertheless, the presence of pluripotency markers even in low quantity suggests that the safety and the absence of tumorigenicity should be thoroughly evaluated for these cells. Our data of paired observations suggest that DPSC and PDLSC are different in their rate of proliferation, pluripotency markers, morphology and osteogenic potential. The data confirm the influence of the niche on the cells of the same origin. DPSC proliferate at a slower rate and have a shape closer to polygonal but they respond better to osteogenic stimuli as compared to PDLSC. *RUNX2* mRNA was detected by qPCR in the both types of dental stem cells but RUNX2 protein was detected by LC-MS/MS shotgun proteomics only in PDLSC suggesting the posttranscriptional regulation. Surprisingly, proteome analysis revealed that RUNX2 was interacting with a lesser number of proteins in osteogenically differentiating PDLSC than in undifferentiated cells while in undifferentiated PDLSC, RUNX2 might be suppressed by histone deacetylases HDAC1 and HDAC2. *DSPP*, a marker gene of odontoblastic type of osteogenic differentiation, was transcribed in DPSC but not in PDLSC samples. Our results prove that DPSC and PDLSC are different in their biology and therapeutic potential: DPSC are a good candidate for osteogenic or bone-replacement cell-seeded medicines while fast proliferating PDLSC are a prospective candidate for other cell products.

## 2. Materials and Methods

### 2.1. Bioethics

All procedures performed in the study involving human beings complied with the ethical standards of the institutional and/or national research ethics committee and the 1964 Helsinki Declaration and its subsequent changes or comparable ethical standards (Declaration of Helsinki: Ethical Principles for Medical Research Involving Human Subjects, including amendments made by the 64th Meeting of World Medical Association in Fortaleza, Brazil, October 2013). An informed written consent was obtained from each of the participants enrolled in the study (or his/her parents for underage).

Children and blastocysts: The ethical committee of Mechnikov North-Western State Medical University approved isolation and expansion in vitro primary PDLSC and DPSC cultures (ethical vote No 12, date of issue 12 December 2019). The local ethical committee of Ava-Peter-Scandinavia assisted reproduction clinics approved the usage of blastocysts in the study (ethical vote #2/23-10-2018, date of issue 23/10/2018)

### 2.2. Cells

Primary cultures of dental stem cells were obtained from donors (median age of the donors—22.1 ± 4.2 years) without systemic diseases and pulp diseases, tooth decay, pulpitis, periodontal disease, denticles.

We obtained successfully 12 pairs (i.e., paired samples from the same tooth of the same donor) of DPSC and PDLSC: twelve DPSC cultures from adult teeth (*n* = 12), and twelve PDLSC cultures (*n* = 12).

Human third molars with residual periodontal ligament were collected from patients during surgical extraction under local anesthesia with articaine (1:200,000). The extraction was performed for medical reasons—dystopia or retention. Teeth with periodontal tissues were transported in isotonic NaCl solution containing 100 U/mL penicillin and 100 μg/mL streptomycin (ThermoFisher Sci, Waltham, MA, USA) at room temperature. The periodontal ligament tissue of the permanent molar root was scraped off with a sterile scalpel and digested in a phosphatx10-buffered saline (PBS) (Life Technologies, Carlsbad, CA, USA) containing 1 mg/mL collagenase type I (ThermoFisher Sci, Waltham, MA, USA), and 1 mg/mL collagenase type IV (ThermoFisher Sci, Waltham, MA, USA) for 40 min at 37 °C in a shaker incubator. The tooth with closed root canals and an inseparable part of ligaments was also placed in a similar solution and incubated for 1 hr. Then, a tooth was removed and the collagenase solution was centrifuged at 400× *g* for 7 min. The pellet was resuspended in a Dulbecco’s modified Eagle, low glucose medium (DMEM LG GlutaMAX, ThermoFisher Sci, Waltham, MA, USA) supplemented with 10% FBS (fetal bovine serum; HyClone, Logan, UT, USA), 100 U/mL penicillin, and 100 μg/mL streptomycin (Life Technologies, USA). The cells were seeded into a flask (TPP, Trasadingen, Switzerland) and were further grown at 37 °C in a humidified 5% CO_2_/7% O_2_ atmosphere. The tooth was transferred after treatment with collagenases into 70% ethanol for 3 min to kill periodontal ligament cells. To obtain DPSC, the internal cavity of the tooth was filled with the collagenase solution through the apical foramen; the tooth was incubated at 37 °C, and then the dissociated pulp was removed through a root channel by pumping saline with a syringe. If the roots were closed or too narrow, the pulp chamber was opened in a sterile environment. The pulp was gently isolated by a syringe needle. The excised pulp tissue was chopped into 1–2 mm^2^ pieces, incubated in collagenases solution, washed and seeded into the cell culture medium as described above for periodontal ligamentum.

Frozen unhatched blastocysts or morulas (5–6 days after in vitro fertilization) stored no less than 5 years were taken for the study if parents decided to donate them for research purposes and declared their decision in written informed consent. The blastocysts were thawed according to the protocol developed by Kitazato Company, incubated in Quinn’s Advantage Blastocyst medium (Cooper Surgical, Trumbull, CT, USA) supplemented with 2% bovine serum albumin (BSA) for 1 h and were further used either for RNA isolation or immunocytochemistry studies as a control sample enriched in pluripotency markers. RNA was isolated using the ExtractRNA kit (Evrogen, Moscow, Russia) and was reverse-transcribed with MMLV RT kit (Evrogen, Moscow, Russia). For immunochytochemistry staining, blastocysts were put on extra-adhesive X-tra Surgipath slides (Leica, Wetzlar, Germany) in a drop of Quinn’s Advantage Blastocyst medium (Cooper Surgical, Trumbull, CT, USA) and in 5 min the remained medium was replaced with 4% formaldehyde in PBS. After 45 min of fixation at room temperature, the cells were permeabilized with 0.05% Triton X-100 in PBS.

### 2.3. Proliferation Rate Assay

PDLSC or DPSC were plated at 1.00 × 10^4^ cells/well. Cells were detached and counted using a cell-counting chamber (Minimed, Bryansk, Russia) and Luna Cell counter (Logos Biosystems, Anyang-si, South Korea) on days 2, 3, 5 and 7 after seeding.

The average number of days between passages was also calculated at each splitting. At each passage, cells were re-plated at the initial density, and subculturing was performed until passage 15.

### 2.4. Immunophenotyping

Cell immunophenotyping was performed using a flow cytometer Navios (Beckman Coulter, Brea, CA, USA) equipped with 2 lasers (488 nm and 638 nm), 8 fluorescence detectors and the original standard set of light filters (Blue Laser: 525/40, 575/30, 620/30, 695/30, 755LP; Red Laser: 660/20, 725/20, 755 LP). The staining and detection were performed according to the standard protocols recommended by the manufacturer.

After the epithelial-mesenchymal transition, neural crest stem cells usually start to express a set of surface markers typical for mesenchymal stem cells (MSC). Therefore, the following monoclonal antibody panels were used to identify positive and negative surface markers typical for MSC [20]: CD44-FITC/CD73-PE/CD90-PC5/CD105-PC7 and CD34-FITC/CD117-PE/CD14-PC5/CD45-PC7 (Beckman Coulter, Brea, CA, USA). Cells were subjected to flow cytometry on the 2nd passage. Autofluorescence level was evaluated using an unstained control sample. The level of non-specific binding of antibodies was determined using isotypic controls (mouse immunoglobulins conjugated to FITC, PE, PC5, PC7). Gating of fluorescence events was carried out using the viability parameter. The viability was estimated by forward and side scattering along with 7-aminoactinomycin D staining. In each sample, at least 15,000 “targeted events” (events determined as viable cells) were analyzed.

### 2.5. The Osteogenic Differentiation of Dental Stem Cells

MSC at passage 3 were seeded at a density of 10^4^ per well into 6 well plates as described above. When cells reached 90–100% confluency, the medium was changed to MSCgo™ Osteogenic (BioInd, Sartorius Group, Göttingen, Germany) for 28 days in order to induce osteogenic differentiation. When the influence of different cell culture conditions was accessed, cells were grown either in Low glucose DMEM or α-MEM supplemented with 10% FBS, 2 mM L-glutamine, 1% penicillin/streptomycin (HyClone, Logan, UT, USA), 50 mg/mL ascorbic acid (Sigma Aldrich, St. Louis, MO, USA), 0.1 mM dexamethasone (Sigma Aldrich, St. Louis, MO, USA) and 10 mM β–glycerophosphate (Sigma Aldrich, St. Louis, MO, USA) either in normoxia (20% O_2_) or in 7% O_2_. Cells were harvested for RNA isolation on days 5, 10. For calcifications staining, cells were fixed with 10% paraformaldehyde (30 min at room temperature) on day 28 and stained with Alizarin Red (Sigma Aldrich, St. Louis, MO, USA) according to a standard protocol.

### 2.6. Real-Time Quantitative PCR (RT-qPCR) Analysis

Total RNA from dental stem cells was isolated using GenElute Mammalian Total RNA Miniprep Kit (Sigma Aldrich, St. Louis, MO, USA). The RNA concentration was measured with a spectrophotometer (NanoQuant Infinite F200 PRO, TECAN). Total RNA (1 µg) was reverse-transcribed with MMLV RT kit (Evrogen, Moscow, Russia). Real-time PCR was performed with 50 ng cDNA and SYBRGreen PCR Mastermix (Evrogen, Moscow, Russia) using CFX96 Real-Time System (Bio-Rad, Hercules, CA, USA). The thermocycling conditions were as follows: 95 °C for 5 min, followed by 45 cycles at 95 °C for 15 s, 60 °C for 30 s and 70 °C for 30 s (a 3-steps protocol is recommended by the PCR master-mix manufacturer). A final heating step of 65 °C to 95 °C was performed to obtain melting curves of the final PCR products. mRNA expression levels were calculated by the 2^−ΔΔCt^ method with the levels of gene transcription normalized to the housekeeping genes *GAPDH* encoding glyceraldehyde 3-phosphate dehydrogenase (GAPDH) and *ACTB* encoding β-actin. Human blastocysts were used as a positive control to evaluate the quantity of *OCT4* mRNA in dental cell cultures. The list of primers used for targeted genes amplification is shown in Table 1.

### 2.7. Immunofluorescence Staining

Cells grown on coverslips (SPL Life Sciences, Pocheon-si, Republic of Korea) were washed twice in PBS, fixed with 4% paraformaldehyde and permeabilized with 0.1% Triton X-100. After blocking overnight with 5% BSA, the cells were incubated with a primary antibody against OCT4/3 (1:200, STEMCELL Technologies, Vancouver, British Columbia, Canada) for 2 h at 37 °C in a humidified 5% CO_2_ incubator. After washing three times in PBS, an Alexa Fluor 488- conjugated secondary antibody (1:200) was added for 1 h. After washing three times in 1× PBS buffer, phycoerythrin-conjugated antibody against SSEA-4 (1:100) (STEMCELL Technologies, Vancouver, British Columbia, Canada) was added for 1 h. Cells were mounted in Slowfade^®^ antifade medium with DAPI (ThermoFisher Sci, Waltham, MA, USA). Human blastocysts enriched in OCT4 and SSEA-4 proteins were used as a positive control for staining.

### 2.8. Microscopy

Image acquisition was performed using an Olympus FV3000 confocal microscope (Nikon, Tokyo, Japan). To detect DAPI, FITC and Cy3, the 405, 488, and 561 nm diode lasers were used for excitation, respectively. The cells were optically sectioned in the *z*-axis with a 0.8 μM interval. Brightness-contrast-intensity correction was applied using the built-in software. Image acquisition for proliferation assay and calcifications estimation was performed with an AxioVert.A1 microscope (Carl Zeiss, Oberkochen, Germany).

### 2.9. Statistical Analysis

All experiments were repeated at least three times. Each experiment was carried out in triplicates. Values are means ± standard deviation. A comparison of mean values between groups was evaluated by a two-tailed Student’s *t*-test using the GraphPad Prism software. *p* values < 0.05 and less were considered as significant.

### 2.10. Proteomics Analysis

DPSCs and PDLSCs at passage 3 were seeded into 90 mm Petri dishes (Eppendorf AG, Hamburg, Germany) and cultured in standard conditions with DMEM (ThermoFisher Sci, Waltham, MA, USA) supplemented with 10% fetal bovine serum (HyClone, Logan, UT, USA), 37 °C, 5% CO_2_. When cells reached 90–100% confluency, the medium was changed to osteogenic medium prepared ex tempore (Low glucose DMEM supplemented with 10% FBS, 2 mM l-glutamine, 1% penicillin/streptomycin (HyClone, Logan, UT, USA), 50 mg/mL ascorbic acid (Sigma Aldrich, USA), 0.1 mM dexamethasone (Sigma Aldrich, USA) and 10 mM β–glycerophosphate (Sigma Aldrich, St. Louis, MO, USA).

Control and differentiated (10th day after induction of osteogenic differentiation) cells were lysed by RIPA lysis buffer (ThermoFisher Sci, Waltham, MA, USA) with SIGMAFAST protease inhibitor cocktail (Sigma Aldrich, St. Louis, MO, USA) then the lysates were collected to microcentrifuge tubes, frozen, sonicated and centrifuged (15 min, 16,000× *g*, 4 °C).

#### 2.10.1. Shotgun Proteomics

Proteins were cleaned by acetone precipitation (EM grade; EMS, Hatfield, PA, USA) and were resuspended in 8M Urea/50 mM ammonium bicarbonate (Sigma Aldrich, St. Louis, MO, USA). Then the protein concentration was measured by Qubit 4.0 fluorometer (Thermo Fisher Sci, Waltham, MA, USA) with QuDye Protein Quantification Kit (Lumiprobe, Moscow, Russia). Protein quantification was verified by protein electrophoresis.

The samples (20 μg) were incubated for 1 h at 37 °C with 5 mM DTT (Sigma Aldrich, St. Louis, MO, USA) with subsequent incubation in 15 mM iodoacetamide for 30 min in the dark at RT (Sigma Aldrich, St. Louis, MO, USA). Next, the samples were diluted with seven volumes of 50 mM ammonium bicarbonate and incubated for 16 h at 37 °C with 400 ng of Trypsin Gold (ratio 1:50; Promega, Madison, WI, USA). A half of each sample was then evaporated in Labconco Centrivap Centrifugal Concentrator (Labconco, Kansas City, MO, USA) and the quality of digestion was verified by protein electrophoresis. The other half of the sample was mixed with formic acid (Sigma Aldrich, St. Louis, MO, USA) to 1% final concentration, evaporated in Labconco Centrivap Centrifugal Concentrator and desalted with C18 ZipTip (MilliporeSigma, Burlington, MA, USA) according to manufacturer recommendations. Desalted peptides were evaporated and dissolved in 20 μL of water/0.1% formic acid for further LC-MS/MS analysis.

Approximate 500 ng of peptides were used for shotgun proteomics analysis by UHPLC-MS/MS with ion mobility in TimsToF Pro mass spectrometer (Bruker Daltonics, Bremen, Germany) with nanoElute UHPLC system (Bruker Daltonics, Bremen, Germany). UHPLC was performed in two-column separation mode with Acclaim™ PepMap™ 5 mm Trap Cartridge (Thermo Fisher Scientific, Waltham, MA, USA) and Bruker Fifteen separation column (C18 ReproSil AQ, 150 mm × 0.75 mm, 1.9 µm, 120 A; Bruker Daltonics, Bremen, Germany) in gradient mode with 400 nL/min flow rate. Phase A was water/0.1% formic acid, phase B was acetonitrile/0.1% formic acid. The gradient was from 2% to 35% phase B for 30 min, then to 95% of phase B for 1 min with subsequent wash with 95% phase B for 5 min. The column was equilibrated with 4 column volumes before each sample. CaptiveSpray ion source was used for electrospray ionization with 1600 V of capillary voltage, 3 l/min N2 flow, and 180 °C source temperature. The mass spectrometry acquisition was performed in automatic DDA PASEF mode with 0.5 s cycle in positive polarity with the fragmentation of ions with at least two charges in *m*/*z* range from 100 to 1700 and ion mobility range from 0.85 to 1.30 1/K0.

Protein identification was performed in Peaks Xpro software (a license granted to St. Petersburg State University; Bioinformatics Solutions Inc., Waterloo, ON, Canada) using human protein SwissProt database (https://www.uniprot.org/; accessed on 17 October 2021; organism: Human [9606]; uploaded on 2 March 2021; 20,394 sequences) and protein contaminants database CRAP (https://www.thegpm.org/crap/; version of 4 March 2019; accessed on 17 October 2021). The search parameters were: parent mass error tolerance 15 ppm and fragment mass error tolerance 0.05 ppm, protein and peptide FDR less than 1%, two possible missed cleavage sites, proteins with at least two unique peptides were included for further analysis. Cysteine carbamidomethylation was set as fixed modification. Methionine oxidation, acetylation of protein N-term, asparagine, and glutamine deamidation were set as variable modifications.

The mass spectrometry proteomics data and protein identification results have been deposited to the ProteomeXchange Consortium via the PRIDE [25] partner repository with the dataset identifier PXD027719 and 10.6019/PXD027719.

Label-free quantification by peak area under the curve was used for further analysis in R (version 3.6.1; R Core Team, 2019). First of all, we performed qualitative analysis—all proteins presented in both biological replicates were identified and the biological groups were compared by Venn diagram with “VennDiagram” package (https://cran.r-project.org/web/packages/VennDiagram/VennDiagram.pdf, accessed on 17 October 2021) [26]. Then the proteins with NA in more than 85% of samples were removed and imputation of missed values by k-nearest neighbors was performed by the “impute” package [27]. Then log-transformation and quantile normalization with further analysis of differential expression by “limma” package were performed [28]. Finally, we performed ordination of samples by principal component analysis and classification of samples by sparse partial least squares discriminant analysis in the package “MixOmics” [29]. “ggplot2” and “EnhancedVolcano” packages were used for visualization [30,31]. Reproducible code for data analysis is available from https://github.com/ArseniyLobov/Proteomic-comparison-of-DPSCs-and-PDLSCs.git (accessed on 17 October 2021).

Functional annotation was performed by the Database for Annotation, Visualization and Integrated Discovery (DAVID) v6.8 (https://david.ncifcrf.gov/, accessed on 26 June 2021; [32]). For the prediction of possible clusters of counteracting proteins, we performed string protein interaction analysis [33].

#### 2.10.2. Gel-Based Proteomics

Two-Dimensional Difference Gel Electrophoresis (2D DIGE) was performed as described earlier [34]. Prior to electrophoresis 35 μg of each sample were conjugated with 400 pM of Cy2, Cy3 or Cy5 fluorophores for 2D electrophoresis according to manufacturer recommendations (Lumiprobe, Moscow, Russia). Then, three samples were mixed and loaded to ready IPG-strip for two-dimensional electrophoresis (pH 3–10, 7 cm, BioRad Laboratories, USA) by passive rehydration overnight at RT in the dark. Separation in the first direction was carried out in a Protean IEF Cell (Bio-Rad, Hercules, CA, USA) using the method recommended by the IPG-strip manufacturer: 10,000 Vh, end voltage 4000 V, rapid ramp, 20 °C. After isoelectric focusing, IPG-strips were sequentially incubated in two equilibration buffers (6 M urea, 2% SDS, 20% glycerin, 0.375 M Tris, pH 8.8) for 10 min in each of them. The first buffer was supplemented with 2% dithiothreitol and the second one—with 2.5% iodoacetamide. The second direction of 2D-electrophoresis was performed in a MiniProtean TetraCell (Bio-Rad, Hercules, CA, USA) in 12% PAAG in Tris/glycine/SDS buffer. Multiplex visualization of different Cy fluorophores was performed in Typhoon FLA 9500 laser scanner (GE Healthcare, Chicago, IL, USA). Each sample was analyzed at least in two technical replicates with different Cy fluorophores.

Raw electropherograms are deposited to the ProteomeXchange Consortium together with shotgun proteomics data with the dataset identifier PXD027719 and 10.6019/PXD027719.

For protein identification, 2D-electrophoresis was repeated similar to 2D DIGE, but with one sample per gel and without Cy fluorophores. For spot excision, gels were stained by Coomassie Brilliant Blue G-250 (Sigma Aldrich, St. Louis, MO, USA).

After excision, proteins were digested by the standard protocol of “in-gel” digestion described earlier [34]. Fragments of PAAG were cut to pieces and washed three times with 50 mM ammonium bicarbonate/50% acetonitrile. Then the gels were dehydrated by acetonitrile, dried up and rehydrated with bovine trypsin solution (20 ng/uL in 50 mM ammonium bicarbonate; Sigma Aldrich, St. Louis, MO, USA). Trypsinolysis was performed at 37 °C overnight. Then tryptic peptides were extracted by 50% acetonitrile/0.1% formic acid, evaporated in Labconco Centrivap Centrifugal Concentrator, and desalted with C18 ZipTip. Protein identification was performed in at least two technical replicates for each protein spot.

Mass spectrometry identification of tryptic peptides from protein spots was similar to shotgun proteomics except for chromatographic separation method: UHPLC was performed with Bruker Ten separation column (C18 ReproSil AQ, 100 mm × 0.75 mm, 1.9 µm, 120 A; Bruker Daltonics, Bremen, Germany) with similar, but shorter gradient (17.1 min). Protein identification was performed in Peaks Xpro with the same parameters as for the shotgun proteomics.

## 3. Results

### 3.1. Morphological Evaluation, Proliferation Rate, Expression of Cell Surface Markers

**Figure 1:**
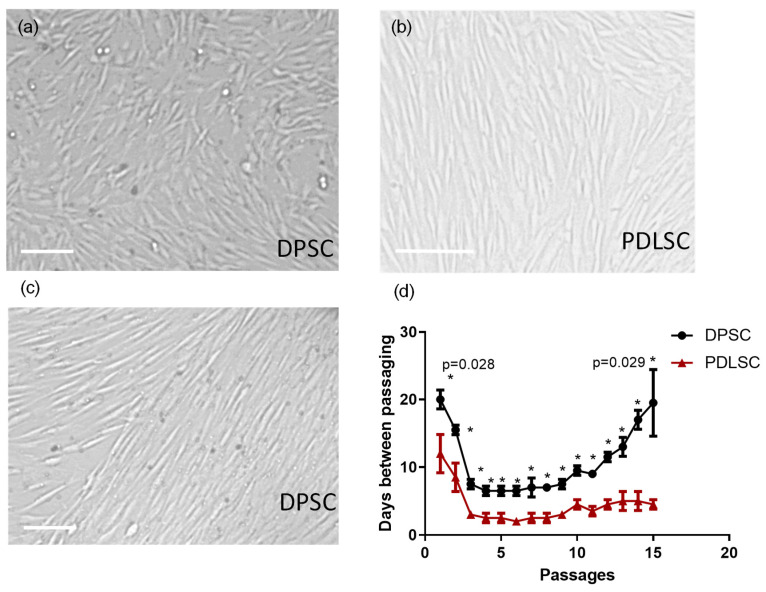
Dental pulp stem cells (DPSC) and periodontal ligament stem cells (PDLSC): morphology (**a**–**c**), proliferation rate and maximal period of growing in vitro (**d**). (**a**) Irregularly shaped DPSC, (**b**) Spindle-like PDLSC, (**c**) Spindle-like DPSC. (**d**) Time between passaging of DPSC and PDLSC cultures. *X*-axis—number of passages, *Y*-axis—days between passages, *—significant (*p* < 0.05) difference between PDLSC and DPSC at the same passage (the exact *p*-value is given for the first and last passages). Scale—50 mkm.

Morphology of cells growing in vitro is an important parameter. Mesenchymal stromal cells and dental stem cells usually have spindle-like fibroblasts morphology at the beginning of expansion in vitro. Spindle-like cells are often organized in groups with the same orientation of ‘spindles’ (i.e., cells’ long axis). Cells from some cultures, especially from those, that proliferate slowly, often have polygonal shape and grow more irregularly. In our study, in 5–6 days after cells isolation, adherent fibroblast-like spindle colonies were observed in all primary cultures. After two passages, PDLSCs as well as DPSCs cultures consisted of fibroblasts-like cells only. PDLSC usually have spindle-like morphology (Figure 1b), while DPSC cultures’ morphology was more variable—in most of the primary cultures, cells had polygonal shape though some had spindle-like morphology (Figure 1a,c). DPSC and PDLSC also differed in their proliferation rate (Figure 1d). The interval before the first passaging was significantly shorter for PDLSC than for DPSC (Figure 1d): 12.0 ± 2.8 vs. 20.0 ± 1,4 (*p* = 0,0001). After continuing cell expansion, DPSC had a lower proliferation rate (passaging frequency 5–7 days) than PDLSC had (passaging frequency—2–3 days). Pulp stem cells were also the first to stop growing in the culture after passage 10, while PDLSC could be passaged 15 or more times (Figure 1d).

Dental stem cells had mesenchymal morphology and immunophenotype (a set of surface markers). The set of MSC cell surface markers [20] on DPSC and PDLSC membranes was analyzed by flow cytometry. Most of the primary stem cells cultures met the MSC criteria established by International Stem Cell Therapy Society. More than 95% of cells were positive for positive MSC markers (CD44, CD90, CD105, CD73) and less than 5% were positive for negative MSC markers (Table 2). However, in both PDSC and PDLSC, a subpopulation of CD117(c-kit)-positive cells was detected. The marker was detected only in cultures at the early passages and disappeared at passage 5 or later.

Thus, both DPSC and PDLSC have mesenchymal stromal cells morphology and the set of surface markers. However, both populations are enriched in CD117 at the beginning of the expansion in vitro. Despite these similarities, PDLSC were the most rapidly proliferating, while DPSC proliferated slowly and quickly ceased to grow in the culture.

### 3.2. Transcription of Nestin (NES) Gene

**Figure 2:**
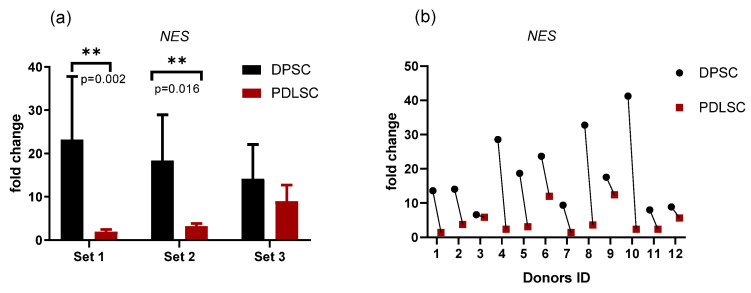
Nestin gene (*NES)* transcription in PDLSC and DPSC quantified by qPCR: comparison between samples obtained from the same donor. (**a**) Average values (mean and standard deviation) obtained in three different experiments (set 1, set 2, set 3; *n* = 4 in each set); (**b**) the values for each donor (donors ID are plotted on the *X*-axis) are shown to demonstrate the variability between donors. *Y*-axis—fold change. The reference gene—*GAPdH*. **—*p* < 0.01 (the exact *p*-values are also shown).

Dental stem cells are very close to MSC in their morphology and surface markers. However, they express ectodermal markers such as a neuroepithelial stem cell protein, Nestin (NES), originally described as a specific glial marker [4,5,35,36]. Now it is assumed that NES is related to essential stem cell functions, including self-renewal/proliferation, differentiation, and migration [37]. Therefore, we measured the level of *Nestin* (*NES*) gene transcription in pairs DPSC/PDLSC from the same donors to access the difference between these populations. The level of *NES* transcription was higher in DPSCs than in PDLSC in all pairs where the transcription was revealed (Figure 2). However, the difference in transcription varied between donors (Figure 2b). Cells from PDLSC/DPSC pairs from two donors did not transcribe *NES* probably due to clonal selection during cell expansion in vitro.

### 3.3. Pluripotency Markers in PDLSC and DPSC

Dental stem cells can express pluripotency markers such as OCT4 and SSEA-4 [7,8,9]. However, it is not known, whether there is any difference between dental stem cells of different origin. Moreover, the *OCT4* gene (*OCT4*) transcription was not quantified against pluripotent stem cells such as blastocyst’s inner mass cells that transcribe *OCT4* at a very high level.

**Figure 3:**
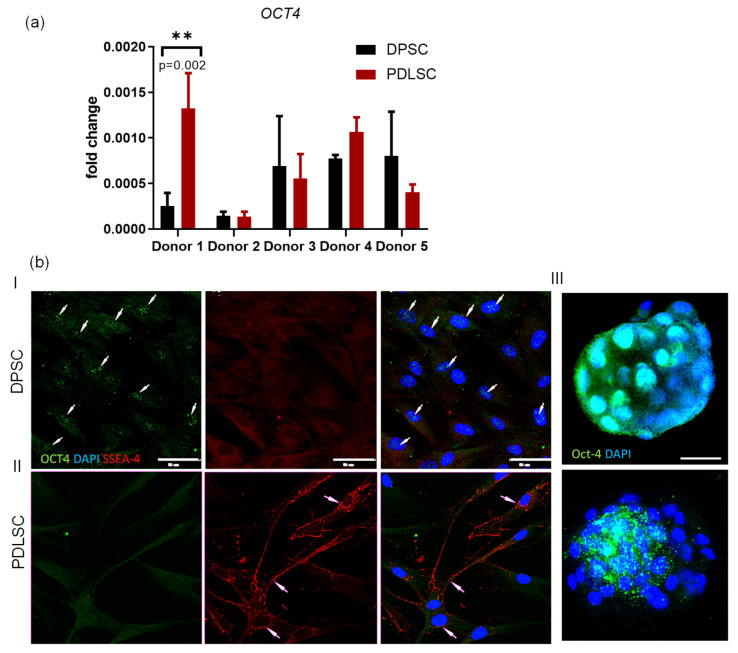
OCT4 and SSEA-4 in DPSC and PDLSC. (**a**) Quantification of *OCT4* mRNA by qPCR. References: GAPdH, β-actin, positive control—blastocyst’s total RNA. **—*p* < 0.01 (the exact *p*-value is also shown); (**b**) Immunofluorescent staining of DPSC (panel I), PDLSC (panel II) with the ABs against OCT4 (green) and SSEA-4 (red). Panel III—a morula (upper image) and a blastocyst (bottom image) stained with the AB against OCT4. According to the previously published data, these two stages are positively stained for OCT4 [38] and were therefore taken as a positive control in our study. Nuclei are counterstained with DAPI (blue). White arrows–positive staining with the AB. Scale bars (50 mkm) are shown in the images.

The presence of *OCT4* RNA in RNA samples was accessed by qPCR with corresponding primers (Table 1). *OCT4* mRNA was detected in cDNA obtained both from PDLSC and DPSC though the level of transcription was very low: 0.0011 ± 0.0004 and 0.0005 ± 0.0001 (Figure 3a) of the level in a blastocyst (its transcription was set as 1). Transcription in dental stem cells varied from 0.0003 to 0.002 of the level in blastocysts. The transcription level of the *OCT4* gene among the DPSCs/PDLSCs paired (taken from the same donor) samples varied both within and between the pairs. In the pair obtained from donor 1, the level of *OCT4* expression in the PDLSCs was 5 times higher than in the DPSCs. One of the pairs showed the same level of *OCT4* expression within the DPSCs/PDLSCs pair but differed from the others by a low level of expression (Figure 3a). Thus, the *OCT4* gene was transcribed both in DPSC and PDLSC though at a very low and variable level.

*OCT4* mRNA was revealed by qPCR (Figure 3a). Earlier the gene transcription was demonstrated in some somatic cells [39,40], but sometimes, the protein was revealed only in the cytoplasm [41,42]. This cytoplasmic protein is not involved in pluripotency maintenance because to establish its function, the protein must act as a transcription factor. Therefore, the OCT4 protein localization was probed by immunostaining with an antibody against it. We also performed staining with an antibody against SSEA-4—another pluripotency marker [43] that had been suggested as a pluripotency marker for living cell flow cytometry because of its exposition on the cell membrane in pluripotent stem cells [44,45,46]. Most of the DPSC (approximately 80%) were positive for OCT4 protein that was localized exclusively in the nucleus interior but not in the cytoplasm (Figure 3b). The cells were not stained with the AB against SSEA-4 (Figure 3b, panel I, red). PDLSC samples were negatively stained with the antiOCT4 AB (Figure 3b, panel II, green). However, SSEA-4 positive signals were revealed in the cytoplasm of approximately 5% of PDLSC. Many signals from the AB delineated the cell membrane (Figure 3b, panel II, red). Both in PDLSC and DPSC, we did not found cells with OCT4 + /SSEA-4+ immunophenotype, which is a feature of adult Very Small Embryonic-Like Stem cells (VSEL) [47] or embryonic stem cells [45,48].

Thus, DPSC and PDLSC do not express pluripotency markers at the same level as pluripotent embryonic stem cells. However, in DPSC, OCT4 is present in nuclei though in small quantity.

### 3.4. Osteogenic Differentiation

**Figure 4:**
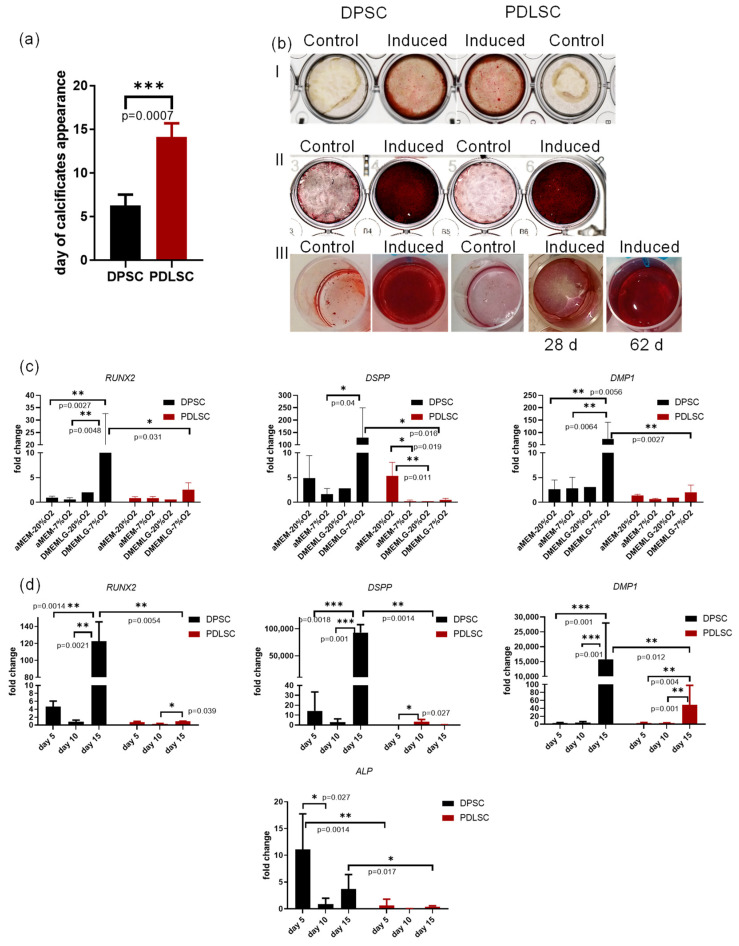
DPSC and PDLSC differentiation after osteogenic induction. (**a**) the rate of appearance of the first visible calcifications, the day when calcifications were revealed is plotted on the *Y*-axis; (**b**) Alizarin staining of DPSC and PDLSC on days 19 (Panel I) and 28 (Panel II) after osteogenic induction. Panel III: a PDLSC sample with delayed differentiation. (**c**) Transcription of osteogenic and odontogenic markers (*RUNX2*, Dentin sialophosphoprotein *DSPP*, Dentin matrix acidic phosphoprotein 1 *DMP1*) after 72 h post-induction in different cell culture conditions. Cells were grown in different O_2_ concentration (hypoxia (7% O_2_) and normoxia, 20% O_2_) and the osteogenic factors were added either in low glucose DMEM or in α-MEM. (**d**) Transcription of osteogenic and odontogenic markers (*RUNX2*, alkaline phosphatase *ALP*, *DSPP*, *DMP1*) during 15 days of differentiation in hypoxia. *Y*-axis—fold change. Reference gene—*GAPdH*. *—*p* < 0.05, **—*p* < 0.01, ***—*p* < 0.001; the exact *p*-values are also shown.

The pluripotency of dental stem cells is still controversial. However, their osteogenic capacity is well-proven [1,10,49,50]. The ability of dental stem cells to respond to osteogenic stimuli either with osteogenic, or cementogenic, or odontogenic differentiation has been demonstrated [49,51]. DMP1 and DSPP, classic odontoblastic markers, are expressed in odontoblasts, dentinal tubules. Their presence is necessary during dentine matrix mineralization [12,35,52]. The osteogenic potential of dental stem cells is probably one of the most important characteristics for their clinical application. Therefore, we studied the rate of osteogenic differentiation, performed a qPCR analysis of osteogenic and odontogenic markers’ transcription in DPSC and PDLSC after osteogenic induction (Figure 4a–d) and compared their proteomes by shotgun proteomics and two-dimensional electrophoresis (see below, Section 3.5). Both populations responded to osteogenic stimuli. On day 20 of incubation in an osteogenic medium, osteogenic differentiation was confirmed by heavy Alizarin red staining (Figure 4b, panels I, II) though one of the PDLSC cell cultures was responding very slowly to the induction (Figure 4b, panel III). DPSC were the fastest responding to osteogenic stimuli—the first calcifications appeared on day 6.25 ± 0.45 while in PDLSC cultures, they were first observed on day 14.10 ± 1.52 (Figure 4a). The delay in response to osteogenic stimuli was confirmed for PDLSC by qPCR (Figure 4c,d). In 72 h after the beginning of osteogenic induction, the mRNA level of *RUNX2* (an early marker of osteogenic/odontogenic differentiation) as well as *DSPP* and *DMP1* (odontogenic differentiation markers) were lower in PDLSC as compared to DPSC. The level of transcription depended on culturing conditions: O_2_ concentration (hypoxia/normoxia) and cell culture medium (DMEM with glucose 1 g/L vs. αMEM). The highest level of transcription was observed in cells cultured in low glucose DMEM in hypoxia conditions (Figure 4c). During the first 15 days of differentiation, the transcription level of *ALP*, *RUNX2*, *DSPP*, *DMP1* was reliably higher in DPSC cells than in PDLSC (Figure 4d). Odontogenic markers and *RUNX2* transcription was increasing faster in DPSC. On day 15, the level of *DMP1* mRNA in DPSC increased 15,807.90 ± 2901.24-fold (X ± m) vs. 49.01 ± 10.1-fold in PDLSC; the level of *DSPP* increased 93,037.99 ± 7314.69-fold in PDSC while in PDLSC, it was downregulated to 0.25 ± 0.04 (Figure 4d).

Thus, both DPSC and PDLSC were capable of osteogenic differentiation. However, DPSC differentiated into the odontoblastic direction.

### 3.5. PDLSCs and DPSCs Have Different Proteomics Profiles during Osteogenic Differentiation

The results of our experiments obtained by qPCR, immunocytochemistry, and microscopy confirmed that DPSC and PDLSC, despite their morphological similarity, represent two different populations of dental stem cells with different functionality. A proteomic comparison was performed for better evaluation of the difference and biological functions of DPSC and PDLSC. For proteomic comparison, we performed shotgun (LC-MS/MS with ion mobility) and gel-based (two-dimensional difference gel electrophoresis; 2D DIGE) analysis of the same samples of PDLSC and DPSC in control and on the 10th day after induction of osteogenic differentiation.

#### 3.5.1. Shotgun Proteomics

**Figure 5:**
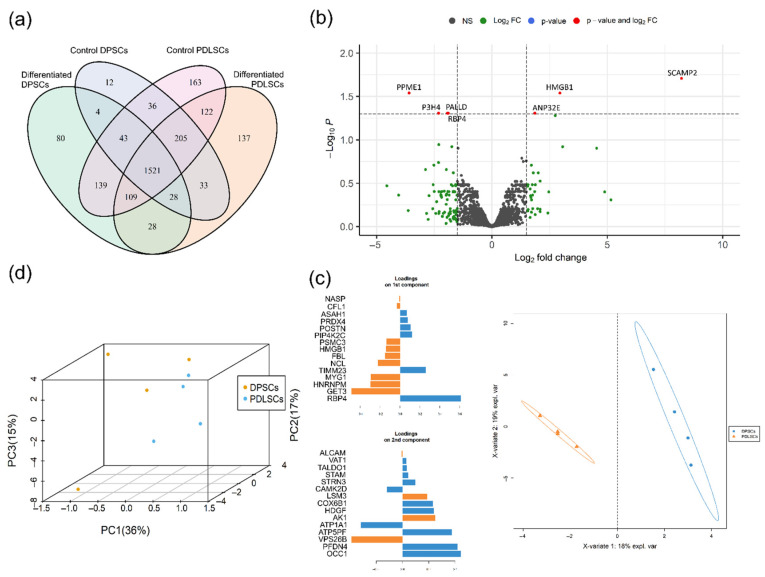
Comparison of proteomics profiles of PDLSC and DPSC during osteogenic differentiation. (**a**) Venn diagram representing proteins unique for biological groups compared by shotgun proteomics. (**b**) Volcano plot representing differentially expressed proteins between DPSC and PDLC. Log2Fold Change—level of change in expression-Log10P—logarithm of *p*–value. Dotted lines cut off transcripts with *p*-value < 0.05 and Log2Fold Change > |1.5|. (**c**) Ordination of DPSC and PDLSC by principal component analysis. Blue dots—DPSC, orange dots—PDLSC. (**d**) Classification of DPSC and PDLSC by sparse Partial Least Squares Discriminant Analysis (sPLS–DA). Blue circles—DPSC, orange triangles—PDLSC. Loadings on the 1st and 2nd component represent 15 proteins, which contribute the most to the respective components, the color represent in which of the group the mean of the protein is maximal.

By the shotgun proteomics analysis using tandem-mass-spectrometry with ion mobility in PASEF mode, we identified 2660 protein groups that have at least two unique peptides and were represented in both biological replicates at least in one biological group. Only 1521 of such proteins were shared among all samples, while 1139 of proteins were unique for some of compared groups, e.g., 422 of proteins were unique for PDLSC and only 96 for DPSC (Figure 5a). The list of accession numbers of proteins unique for PDLSC and DPSC is given in the Supplemented Materials (File S1).

Enrichment analysis against GO biological process database (GO BP) revealed, that proteins, unique for differentiated DPSCs (80 proteins) are involved in protein transport (*p*-Value = 8.4 × 10^−3^), cytoskeleton organization (*p*-Value = 7.7 × 10^−4^), extracellular matrix organization (*p*-Value = 1.1 × 10^−2^), intracellular protein transport (*p*-Value = 2.1 × 10^−2^), cell-to-cell adhesion (*p*-Value = 3.3 × 10^−2^), regulation of focal adhesion assembly (*p*-Value = 2.6 × 10^−3^) and vesicle docking involved in exocytosis (*p*-Value = 6 × 10^−3^). According to these biological process, enrichment analysis against KEGG database revealed proteins associated with focal adhesion/regulation of actin cytoskeleton (*p*-Value = 6.4 × 10^−3^ and 7 × 10^−3^, respectively) and endocytosis (*p*-Value = 5 × 10^−2^).

Control (undifferentiated) and differentiated DPSC together have only four unique proteins (HSPG2, SEC24A, TMEM106B, VNN1). All four of them were described in the extracellular vesicles and therefore might be associated with extracellular matrix organization. HSPG2 expression is activated in the dental pulp when orthodontic force is applied. The protein is important in repairing and remodeling ECM in tissue stroma and basement membrane [53].

Proteins specific for control (undifferentiated) PDLSC (163 proteins) are associated with many biological processes among which cell cycle and DNA replication (e.g., Cell cycle and DNA replication, KEGG, *p*-value = 9.2 × 10^−4^ and 8.0 × 10^−5^), splicing (e.g., mRNA splicing, via spliceosome, GO BP, *p*-Value = 3.6 × 10^−3^), cell proliferation (e.g., positive regulation of cell proliferation, GO BP, *p*-Value = 8.3 × 10^−3^) and negative regulation of apoptotic process (GO BP, *p*-Value = 2.10 × 10^−2^). Control PDLSC express a number of unique metabolic proteins involved in KEGG “Metabolic pathways” (*p*-Value = 2.7 × 10^−2^) with associated biological processes: purine ribonucleoside monophosphate biosynthetic process (*p*-Value = 5.6 × 10^−3^), nucleotide biosynthetic process (*p*-Value = 7.5 × 10^−3^), positive regulation of collagen biosynthetic process (*p*-Value = 1.7 × 10^−2^), response to nutrient (*p*-Value = 2.8 × 10^−2^), regulation of glucose transport (*p*-Value = 3.4 × 10^−2^), oxidation-reduction process (*p*-Value = 3.7 × 10^−2^), response to nutrient (*p*-Value = 2.8 × 10^−2^), regulation of glucose transport (*p*-Value = 3.4 × 10^−2^), cellular response to retinoic acid (*p*-Value = 2.4 × 10^−2^) and cholesterol biosynthetic process (*p*-Value = 4.4 × 10^−2^). Finally, there are some proteins associated with signaling pathways and cell differentiation: wound healing (*p*-Value = 7.4 × 10^−5^), positive regulation of tyrosine phosphorylation of Stat3 protein (*p*-Value = 4.4 × 10^−2^), ATP-dependent chromatin remodeling (*p*-Value = 1.0 × 10^−3^), planar cell polarity pathway involved in neural tube closure (*p*-Value = 4.8 × 10^−3^), protein sumoylation (*p*-Value = 2.0 × 10^−2^), activation of GTPase activity (*p*-Value = 3.3 × 10^−2^), chromatin remodeling (*p*-Value = 4.0 × 10^−2^), peptidyl-tyrosine phosphorylation (*p*-Value = 4.7 × 10^−2^). Among these proteins WNT5A, WNT5B HDAC1, HDAC2, AKT2 should be emphasized as proteins associated with osteogenic differentiation (more information in the discussion section).

Differentiated PDLSC have 137 unique proteins involved in apoptosis (e.g., apoptotic process, GO BP, *p*-Value = 2.4 × 10^−3^) and cell-cell adhesion (GO BP, *p*-Value = 4.9 × 10^−2^)

Interestingly, RUNX2—the main transcriptional factor of osteogenic differentiation—was found only in both control and differentiated PDLSC, while it was under detection range in all DPSC samples. The results differed from those obtained by qPCR (Figure 4c,d) probably due to DDA mass spectrometry limitations, delayed expression of transcribed mRNA or post-translational regulation of RUNX2. Another possible explanation is posttranscriptional downregulation of RUNX2 during osteogenic differentiation in the time-point selected for proteomics analysis (10th day) [54]. A similar pattern was observed for Akt1 and CDK1—proteins associated with cell differentiation decisions and osteogenic differentiation.

After qualitative analysis, only proteins found in more than 85% of samples were used for quantitative analysis (1830 proteins). We started from the ordination of samples by Principal Component Analysis (PCA) and classification by sparse Partial Least Squares Discriminant Analysis (sPLS-DA). Both methods revealed two distinct clusters of DPSC and PDLSC—these cells have different proteomic profiles before and during osteogenic differentiation (Figure 5c,d). Using sPLS-DA we found 30 proteins with the higher contribution to the observed classification pattern, 15 of which were contributed to the first component with the biggest resolution between DPSC and PDLSC (Figure 5c). Thus, we might indicate proteins with higher abundance in DPSC (ASAH1, PRDX4, POSTN, PIP4K2C, TIMM23, RBP4) and in PDLSC (NASP, CFL1, PSMC3, HMGB1, FBL, NCL, MYG1, HNRNPM, GET3).

Further, we performed differential expression analysis and compared all DPSC and PDLSC and found seven differentially expressed genes (DEGs) with Fold change higher than 1.5 and adjusted *p*-value less than 0.05 (Figure 5b). Four of DEGs are more abundant in DPSC (PPME1, P3H4, RBP4, PALLD) and three more abundant in PDLSC (SCAMP2, HMGB1, ANP32E). Two of the proteins were identified by sPLS-DA and differential expression analysis: RBP4 (more abundant in DPSC) and HMGB1 (more abundant in PDLSC).

#### 3.5.2. Gel-Based Proteomics

**Figure 6:**
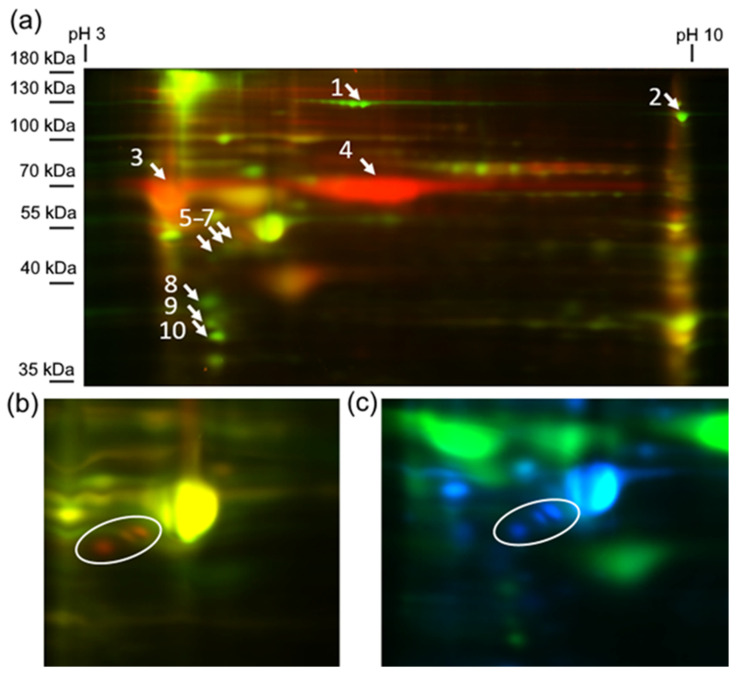
Differentially expressed proteins identified by 2D–DIGE. (**a**) Electropherogram corresponding to the overlapping of Cy fluorochrome channels of control (green) and differentiated (red) DPSC. Numbers marked differentially expressed protein spots reproducibly presented in both biological replicates of some of the biological groups. (**b**) Electropherogram corresponding to the overlapping of Cy fluorochrome channels of control (green) and differentiated (red) PDLSC. White circle—protein spots 5–7, specific for differentiated PDLSC. (**c**) Electropherogram corresponding to the overlapping of Cy fluorochrome channels of differentiated DPSC (green) and differentiated (blue) PDLSC. White circle—protein spots 5–7, specific for differentiated PDLSC.

Gel-based and shotgun proteomics are known to be complementary. Thus, in addition to shotgun proteomics, we performed gel-based analysis by 2D DIGE. Among 240 spots identified in electropherograms we found 10 differentially expressed protein spots marked in Figure 6 (fragments of electropherograms with comparison of marked spots are presented in the supplementary Figure S1; full raw electrophoregrams are available on ProteomeXchange, PXD027719).

These 10 protein spots were identified by MS/MS (Table 3). Spots number 1, 2, 8, 9, and 10 were downregulated during differentiation of both cell types. Spots 1 and 2 were identified as collagen alpha-1(I) and alpha-2(I) chains respectively; spots number 8 and 10 as Tropomyosin beta and alpha-1 chains; spot 9 as Annexin A2.

Several cell-type-specific proteins were also identified. Spots number 5–7, identified as vimentin, were upregulated in differentiated PDLSC, while spots 3 and 4 were specific for differentiated DPSC. Spot 3 was identified as Prelamin-A/C, but we found at least four proteins reproducibly identified in spot 4: Annexin A6, Heat shock cognate 71 kDa protein, Cytoskeleton-associated protein 4, Lamin-B2 (Table 3).

By the shotgun proteomics, we found dozens of cell-type-specific proteins (Figure 5) which involved in many biological processes. PDLSCs had more unique proteins compared to DPSCs. Most of the PDLSCs-specific proteins were associated with the cell cycle, proliferation, and metabolism. The data are in good accordance with the higher proliferative and migration activity of PDLSCs while DPSCs might be considered as more committed to ECM production. DPSCs-specific proteins are associated with protein transport, extracellular matrix organization, exocytosis, etc.

**Figure 7:**
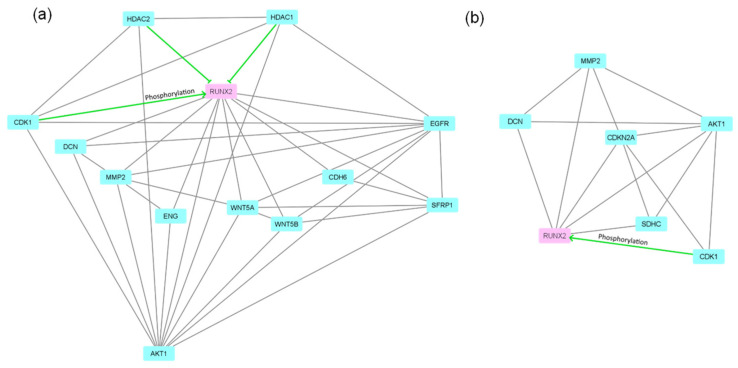
String interaction networks of proteins interacting with RUNX2 among PDLSCs-specific proteins founded in control cells (**a**) and cells after induction of osteogenic differentiation (**b**). Green—protein-protein interactions with experimental evidence. The image was obtained in the Cytoscape software v. 3.8.1 (Cytoscape Consortium; https://cytoscape.org/; accessed on 21 July 2021).

Nevertheless, RUNX2—a key master protein of osteogenic differentiation was exclusively found in both control and differentiated PDLSCs while it was out of a detection range in all DPSCs samples. Thus, phenomena might be an artifact of DDA proteomics or have biological nature. To evaluate possible differences in RUNX2 regulation we analyzed protein interaction networks (by String database; string-db.org; accessed on 20 July 2021) of proteins capable of interaction with RUNX2. We analyzed proteins unique for PDLSCs in control (overall unique proteins plus proteins unique for control PDLSCs; Figure 7a) and after induction of osteogenic (overall unique proteins plus proteins unique for differentiated PDLSCs; Figure 7b). Surprisingly we found a relatively small number of proteins interacting with RUNX2. Moreover, most interactions were predicted by indirect evidence. CDK1, AKT1, EGFR, and some other proteins were able to interact with dozens of proteins in the tested dataset (data not shown). Thus, RUNX2 is in the periphery of the PDLSCs-specific protein interaction network.

According to mass-spectrometry data, the proteomes of DPSC and PDLSC are different. PDLSC proteomes are enriched for proteins, responsible for cell cycle control, proliferation, secretion.

## 4. Discussion

The present study compared the morphological properties, pluripotency markers expression, osteogenic/odontogenic potential, and proteomes of PDLSC and DPSC. The two populations differed in each of these parameters. We used DPSC-PDLSC pairs obtained from the same donor. Paired observations led us to the conclusions that the variability between donors is high, though general trends are similar in most of the pairs. In our study, DPSC were characterized as relatively slow-proliferating cells (especially at the beginning of in vitro expansion) with the MSC set of surface markers, low-level expression of OCT4 and the ability to differentiate into the odontogenic direction. PDLSC proliferated faster, did not express OCT4, but were positive for SSEA-4 and were capable for general osteogenic but not odontogenic differentiation.

Some authors report that PDLSC proliferate more slowly than DPSC [6,8,55], while others describe slow, fast, and intermediate proliferation rates in different samples of PDLSC [56]. The existence of fast and slowly proliferating DPSC subpopulations has also been reported [57]. All these studies have been carried out on cells grown in normoxia (20% O_2_) that is a non-physiological O_2_ concentration for cells in primary cultures. For most tissues, the physiological O_2_ concentration does not exceed 8% [58,59]. MSC grown permanently in “physiological hypoxia” conditions have increased proliferation rate, OCT4 expression, chondrogenic potential [59]. A similar tendency has been demonstrated for dental stem cells though some of the authors admit our limited knowledge on this issue [8,60,61,62]. Therefore, in our studies, the slow rate of DPSC proliferation might be explained by the greater survival of the slow-proliferating clones at the physiological hypoxia.

The set of DPSC and PDLSC surface markers corresponded to the set of markers of MSC. However, in cells of both origins, a CD117 (c-kit) positive subpopulation of stem cells was identified (Table 2). CD117-positive DPSC are considered as less differentiated subpopulation [63,64]. Moreover, some of these DPSC cells expose CD34 on their surface. These cells showed a slower proliferation, gradual loss of stemness, early cell senescence, and apoptosis [57]. c-Kit is a marker of dental pulp progenitor cells and is involved in DPSC self-renewal and stemmness maintenance [65,66,67]. The protein is also expressed in PDLSC [66]. On the other hand, staining for CD117 occurs in a variety of tumor types, although strong staining is present mainly in mast cell disease and gastrointestinal stromal tumors, for which CD117 is the preferred marker [68,69]. Given the c-kit as well the Oct-4 expression along with the fast proliferation, the issue of biological safety of dental stem cells must be thoroughly studied.

*NES* (Nestin) gene was transcribed at a significantly higher level in DPSC than in PDLSC in all the donors (Figure 2). DPSC are known to derive from neural crest cells and are inclined to differentiate into neural cells [36]. DPSC have a higher positive ratio for neural markers such as NES, GFAP, and s100-beta than other kinds of MSC [5,36,70,71]. However, PDLSC have different embryonic origin: dental pulp is formed from dental papilla while PDLSC originate from dental follicle cells [7,72]. NES is considered as a marker not only of DPSC but also of odontoblasts and denticle lining cells, suggesting that denticle cells and odontoblast-like cells may derive from the same pulp stem cell populations [35]. Taking this into account, a greater tendency of DPSC to odontogenic differentiation in comparison with the PDLSC (Figure 4c,d) can be expected.

In our study, staining with an OCT4 antibody revealed the protein only in DPSC nuclei while SSEA-4 positive signals were revealed in the PDLSC cytoplasm only (Figure 3b). According to quantification data, the *OCT4* gene transcription level was very low in DPSC and PDLSC as compared to embryonic stem cells of blastocysts: transcription in dental stem cells varied from 0.0003 to 0.002 of the level in blastocysts (Figure 3a). The low quantity of transcripts might explain the absence of PDLSC staining with the AB against OCT4—the quantity of the protein expressed from the mRNA is probably below the detection limit. OCT4, also known as POU5F1, is a nuclear transcription factor that is necessary for the maintenance of the pluripotency of stem cells and primordial germ cells [73]. It is also a diagnostic marker of some pluripotential germ cell tumors as dysgerminoma and embryonal carcinoma and for in situ germ cell neoplasia such as intratubular germ cell neoplasia in the testis and gonadoblastoma in dysgenetic gonads [68]. There are tumors in which the expression of the *OCT4* gene is increased, but its activation is associated with the movement of the gene under the active promoter, but not with the mechanisms involved in embryonic cells [74]. Ectopic expression of OCT4 in certain somatic cells has been associated with active dedifferentiation [75] or some other effect e.g., atheroprotection [39]. It is also transcribed in MSC at low passages [76]. This finding suggests that it plays a key role not only in maintaining the pluripotency of embryonic stem cells but also in self-renewal and protection against apoptosis of somatic stem cells and tumor-initiating cells. However, researchers from the Dr. R. Jaenish group argued against the role of OCT4 in self-renewal, proliferation and pluripotency maintenance [77]. The controversy might be explained by the fact that *OCT4* generate three main protein isoforms: OCT4A, OCT4B [78], and OCT4B1 [79]. Most studies have focused on the OCT4A as a transcription factor responsible for stemness properties. The 360-amino-acid OCT4A protein is the gatekeeper to pluripotency, the other variants have been associated with antiapoptotic effects and stress responses, but they do not share the pluripotency characteristics of OCT4A [80]. The OCT4 primer set used in this study detects all main isoforms of the transcripts [24]. In our study, the level of OCT4 transcription was 1000–10,000-fold less than in blastocyst’s cells when probed by qPCR. These results suggested that either a percentage of pluripotent stem cells was very low in the samples or, if the protein was present in many nuclei but in low quantities (Figure 3), that it can have other functions in dental stem cells. OCT4 is involved in the maintenance of MSC characteristics in DPSC [81]. The depletion of OCT4 decreased the proliferation and osteogenic properties of DPSC, while overexpression of OCT4 enhanced the proliferation rate and osteogenic/chondrogenic/adipogenic potential of DPSC. The expression of OCT4, SSEA-4 and other ES markers in human PDLSC were described earlier [82,83]. The exposition of SSEA-4 on the cell surface is considered as one of the markers of pluripotent cells [43] suitable for cell sorting when OCT4 staining is not possible [44,45,46]. Nevertheless, it is also expressed in a line of immortalized bone marrow MSC and in a subpopulation (1–2%) of non-transformed primary bone marrow MSC [84]. SSEA-4 is known as a marker of PDLSC [9]. We demonstrated for the first time that DPSC and PDLSC are different in their pluripotency markers levels. Besides, transcription and expression of OCT4 and SSEA-4 are not always coupled in the same cell.

In our study, both DPSC and PDLSC were capable of osteogenic differentiation and deposition of Alizarin Red stained calcifications. However, it has been shown that extracellular matrix produced by different population of dental stem cells varies in its composition though all variations were stained by Alizarin Red [10]. Our data prove the difference between two populations of dental stem cells in their mechanisms of osteogenic differentiation. We observe odontoblastic markers only in samples differentiated from DPSC. DPSC are known to be capable of odontoblastic differentiation [14,15,55] and are also responsible for tertiary dentin formation [12] and denticle biomineralization [35]. PDLSC mechanism of osteogenic differentiation is not the same as in DPSC. They can differentiate into cementoblasts, whose biological function is cementogenesis aimed to provide the anchoring of the periodontal ligament to the tooth [12]. We have demonstrated earlier that human PDLSC differentiation after osteogenic induction is promoted by Notch [85], while DPSC odontoblastic differentiation is inhibited by this pathway [86]. Nevertheless, PDLSC are involved in oral cavity regeneration processes. Prof. T. Inoue’s group demonstrated that it was PDLSC but not mesenchymal stem cells and hematopoietic stem cells of the bone marrow that were involved in the regeneration of the periodontium [87]. Some authors have observed odontoblastic differentiation of PDLSC [11]. In our work, we tried to exclude the mixing of PDLSC and DPSC—a tooth was treated with ethanol to kill the cells on its surface (residual ligament, apical papilla) before opening the pulp chamber. In such conditions, we did not observe odontogenic differentiation of PDLSC.

To study further the overall functional similarity and differences of DPSCs and PDLSCs we performed their untargeted proteomics analysis by two complementary approaches: 2D-DIGE (gel-based proteomics) and Label-free shotgun proteomics with ion mobility. We found major differences between differentiated DPSCs and PDLSCs by both methods (Figure 5 and Figure 6). DPSCs and PDLSCs form distinct clusters on both PCA (ordination) and sPLS-DA (classification), which confirms our assumption of physiological differences existing between DPSCs and PDLSCs before and after osteogenic differentiation. We identified several groups of cell-type-specific proteins. In the two-dimensional electrophoresis, we identified vimentin as exclusively upregulated during osteogenic differentiation of PDLSCs and Prelamin-A/C, Lamin-B2, Annexin A6, Heat shock cognate 71 kDa protein and Cytoskeleton-associated protein 4 as unique for differentiated DPSCs.

Vimentin is a mesenchymal intermediate filament protein. This protein has structure function, but it is also known to be involved in cell proliferation and differentiation [88,89]. Interesting that we might detect at least three spots identified as vimentin (Figure 6)—these protein spots probable correspond to different vimentin isoforms or specific post-translational modification, but we have not found significant differences in MS/MS identification of these spots.

Lamins are nuclear intermediate filaments tightly associated with mechanotransduction influenced cell differentiation and migration [90]. Particularly, the level of Lamins-A/C is known to be increased during osteogenic differentiation [91]. The higher abundance of vimentin and the lower level of lamins in PDLSCs might be associated with the higher migratory and proliferative activity of these cells. In opposite, DPSCs seems to be less proliferative, but secreting more ECM. According to these, they have a higher amount of lamins, Heat shock cognate 71 kDa protein (protein quality control), Cytoskeleton-associated protein 4 (antiproliferative receptor in epithelial cells, structure component of endoplasmic reticulum) and Annexin A6 (involved in exocytosis and ECM mineralization).

By the shotgun proteomics, we found dozens of cell-type-specific proteins (Figure 5), which are involved in many biological processes. PDLSCs have more unique proteins compared to DPSCs. Most of the PDLSCs-specific proteins are associated with the cell cycle, proliferation and metabolism which is in good accordance with the higher proliferative and migration activity of PDLSCs while DPSCs might be considered as more committed to ECM production. This observation can explain our data about faster proliferation of PDLSC as compared to DPSC. DPSCs-specific proteins are associated with protein transport, extracellular matrix organization, exocytosis, etc.

RUNX2—a key master protein of osteogenic differentiation—was exclusively found in both control and differentiated PDLSCs while it was out of a detection range in all DPSCs samples. RUNX2 is known to be unnecessary for osteogenic differentiation of dental follicle cells [92]. Moreover, in the tumor cells, RUNX2 is known to be associated with increased cell migration and proliferation, but not with osteogenic differentiation [93,94]. Particularly, melanoma cells with RUNT-deleted form of RUNX2 have reduced proliferation, increased apoptosis, and reduced EMT [94]. Finally, it was shown that RUNX2 regulated osteoblast proliferation and was necessary for normal cell cycle progression [95]. Thus, the presence of RUNX2 in both control and differentiated PDLSCs might be interpreted in the context of higher migratory and proliferative activity of PDLSCs. In DPSC, RUNX2 is transcribed though the level of transcription strongly depended on cell culture conditions (Figure 4c,d). Nevertheless, the protein was not detected by mass-spectrometry in any DPSC samples. It has been demonstrated that the effect of RUNX2 during odontoblast differentiation is stage-dependent. RUNX2 inhibits odontoblast terminal differentiation and induces transdifferentiation of odontoblasts to osteoblasts at the late cell differentiation stage [96,97,98]. Therefore, we suggest that the RUNX2 gene can be transcribed in DPSC but the mRNA translation might be delayed or stopped. The post-translational regulation of this protein [54] may also be an important factor.

## 5. Conclusions

Our results prove that DPSC and PDLSC are different in their biology and therapeutic potential: DPSC are a good candidate for osteogenic or bone-replacement cell-seeded medicines while fast proliferating PDLSC are a prospective candidate for other cell products.

## Figures and Tables

**Table 1:** List of primers used for quantification of targeted genes transcription.

Encoded Protein	Primer Sequence	
Alkaline Phosphatase (ALP) forward [21]	5′-TCAGAAGCTCAACACCAACG-3′	A marker of osteogenic differentiation
Alkaline Phosphatase (ALP) reverse [21]	5′-GTCAGGGACCTGGGCATT-3′	
Runt-related transcription factor 2 (Cbfa-1/RUNX2) forward [22]	5′-CCGCCTCAGTGATTTAGGGC-3	An early marker of osteogenic differentiation
Runt-related transcription factor 2 (Cbfa-1/RUNX2) reverse [22]	5′-GGGTCTGTAATCTGACTCTGTCC-3	
Dentin Sialophosphoprotein (DSPP) forward (own design)	5′-TCAGAGACACATGCTGTTGGG	A dentin protein, a marker of odontoblastic differentiation
Dentin Sialophosphoprotein (DSPP) reverse (own design)	5′-CTTTACCTTCGTTGCCTTTCCC-3	
Dentin Matrix Acidic Phosphoprotein 1 (DMP1) Forward (own design)	5′-TCTTTGTGAACTACGGAGGGT-3	A marker of odontoblastic differentiation
Dentin Matrix Acidic Phosphoprotein 1 (DMP1) Reverse (own design)	5′-CCTGGTTACTGGGAGAGCAC-3	
Nestin forward [23]	5′-GCGTTGGAACAGAGGTTGGA-3′	A marker of stem cells of neural crest origin
Nestin reverse [23]	5′-TGGGAGCAAAGATCCAAGAC-3′	
OCT4 forward [24]	5′-ACATCAAAGCTCTGCAGAAAGA-3′	A pluripotency marker
OCT4 reverse [24]	5′-AATACCTTCCCAAATAGAACCC-3′	
glyceraldehyde 3-phosphate dehydrogenase (GAPDH) forward (own design)	5′-AGGTCGGAGTCAACGGATTT-3′	Reference genes
glyceraldehyde 3-phosphate dehydrogenase (GAPDH) reverse (own design)	5′-TTCCCGTTCTCAGCCTTGAC-3′	
β-actin (ACTB) forward [21]	5′-ATTGCCGACAGGATGCAGA-3′	
β-actin (ACTB) reverse [21]	5′-GAGTACTTGCGCTCAGGAGGA-3′	

**Table 2:** The number of cells expressing positive and negative surface markers of MSC in cell cultures (passage 2) obtained from the dental pulp of permanent teeth (DPSC) and the periodontal ligament (PDLSC).

Surface Antigens		DPSC	PDLSC
Positive markers of MSC	CD44	98.5 ± 0.65	97.4 ± 0.5
	CD73	97.9 ± 0.61	98.8 ± 0.9
	CD90	99.0 ± 0.69	97.4 ± 0.71
	CD105	98.5 ± 0.72	97.2 ± 0.62
Negative markers of MSC	CD14	1.2 ± 0.05	2.1 ±0.81
	CD34	0.84 ± 0.31	0.42 ± 0.21
	CD45	2.1 ± 0.92	1.45 ± 0.56
	CD117	17.12 ± 2.3	21.0 ± 1.8

**Table 3:** Results of MS/MS identification of protein spots from Figure 6. The best scores from at least two technical replicates are presented.

№ of Protein Spot	UniProt Accession	Protein Name	Peaks Xpro Dentification Probability (−10lgP)	Protein Coverage, %	Number of Unique Peptides	MW, kDa	Comment
1	P02452	Collagen alpha-1(I) chain	525.21	66	69	138,942	Downregulated in differentiation of both cell types
2	P08123	Collagen alpha-2(I) chain	451.38	28	26	129,314	Downregulated in differentiation of both cell types
3	P02545	Prelamin-A/C	255.33	31	23	74,140	Upregulated in differentiated DPSC
4	P08133	Annexin A6	416.45	71	51	75,873	Upregulated in differentiated DPSC
	P11142	Heat shock cognate 71 kDa protein	393.45	59	30	70,898	
	Q03252	Cytoskeleton-associated protein 4	337.48	55	29	66,023	
	Q07065	Lamin-B2	320.73	47	27	69,948	
5	P08670	Vimentin	403.16	75	43	53,652	Upregulated in differentiated PDLSC
6	P08670	Vimentin	352.72	68	40	53,652	Upregulated in differentiated PDLSC
7	P08670	Vimentin	396.21	71	70	53,652	Upregulated in differentiated PDLSC
8	P07951	Tropomyosin beta chain	299.34	53	6	32,851	Downregulated in differentiation of both cell types
9	P07355	Annexin A2	283.10	37	12	38,604	Downregulated in differentiation of both cell types
10	P07951	Tropomyosin alpha-1 chain	284.12	43	15	32,709	Downregulated in differentiation of both cell types

## Data Availability

The mass spectrometry proteomics data have been deposited to the ProteomeXchange Consortium via the PRIDE [25] partner repository with the dataset identifier PXD027719 and 10.6019/PXD027719.

## Supplementary Materials

The following are available online at https://www.mdpi.com/article/10.3390/biomedicines9111606/s1, File S1: Accesion No of identified proteins, Figure S1: Fragments of raw electropherograms corresponding to protein spots described in the main text of Kotova et al., 2021.
