# Supplementary material for: Comparative Analysis of Dental Pulp and Periodontal Stem Cells: Differences in Morphology, Functionality, Osteogenic Differentiation and Proteome
Source: Biomedicines. 2021 Nov 3;9(11):1606. doi: 10.3390/biomedicines9111606 (PMC8616025; doi:10.3390/biomedicines9111606)
Supplement: Supplementary file 1 [file biomedicines-09-01606-s001.zip › Figure S1.pdf]

Supplementary Materials. Figure S1. Fragments of raw electrophoregramms corresponding to protein spots described in the main text of Kotova et al., 2021

Number	Proteins identified	First donor				Second donor			
		Differentiated DPSCs	Control DPSCs	Differentiated PDLSCs	Control PDLSCs	Differentiated DPSCs	Control DPSCs	Differentiated PDLSCs	Control PDLSCs
1	Collagen alpha-1(I) chain	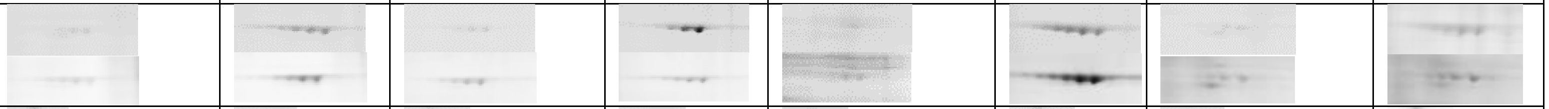							
2	Collagen alpha-2(I) chain	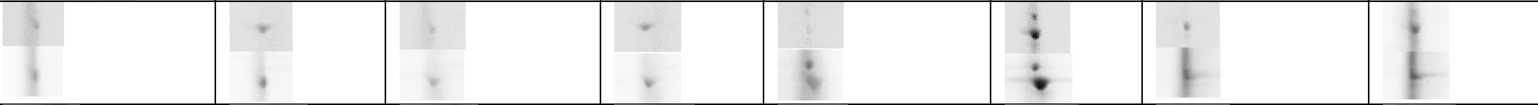							
3	Prelamin-A/C	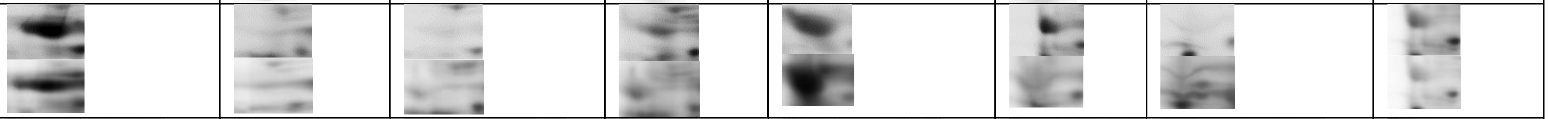							
4	Annexin A6<br>Heat shock cognate 71 kDa protein<br>Cytoskeleton-associated protein 4<br>Lamin-B2	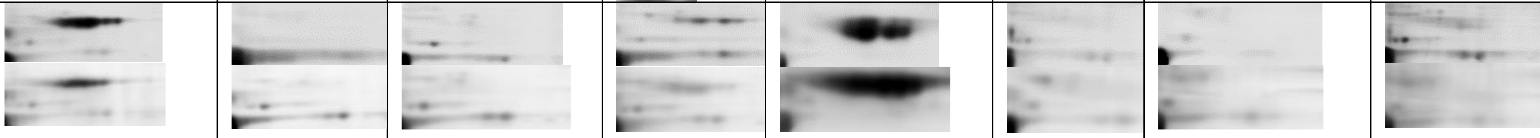							
5, 6, 7	Vimentin	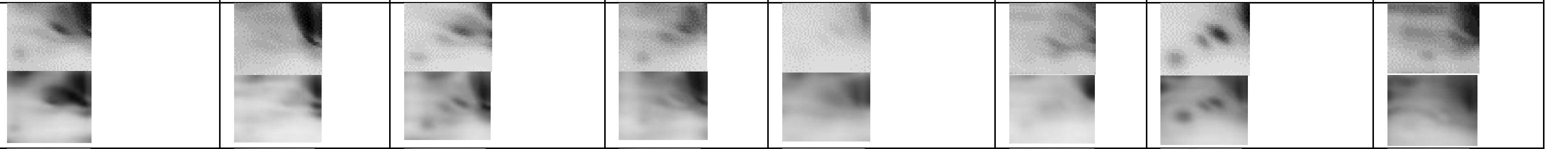							
8	Tropomyosin beta chain	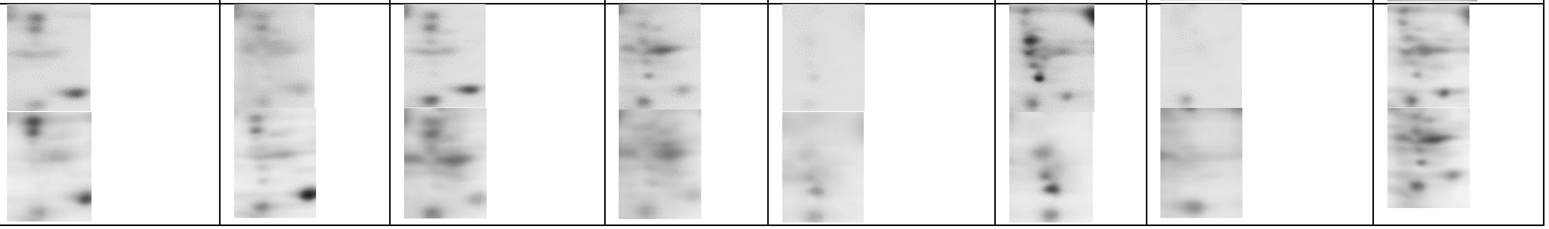							
9	Annexin A2								
10	Tropomyosin alpha-1 chain								
